# Associations between Work Activity and Work Setting Categories and Dimensions of Pharmacists’ Quality of Work Life

**DOI:** 10.3390/pharmacy6030062

**Published:** 2018-07-04

**Authors:** Jon C. Schommer, Caroline A. Gaither, William R. Doucette, David H. Kreling, David A. Mott

**Affiliations:** 1 College of Pharmacy, University of Minnesota, 308 Harvard Street, S.E., Minneapolis, MN 55455, USA; cgaither@umn.edu; 2 College of Pharmacy, University of Iowa, S518 PHAR, Iowa City, IA 52242, USA; william-doucette@uiowa.edu; 3 School of Pharmacy, University of Wisconsin—Madison, 777 Highland Avenue, Madison, WI 53705, USA; david.kreling@wisc.edu (D.H.K.); david.mott@wisc.edu (D.A.M.)

**Keywords:** pharmacist workforce, quality of work life

## Abstract

The goal for this study was to use data from the most recently conducted National Pharmacist Workforce Survey to compare eight components of quality of work life for actively practicing pharmacists in the United States categorized by (1) work activity and (2) work setting. The eight components of quality of work life were: (1) time stress; (2) responsibility stress; (3) level of control; (4) work in harmony with home life; (5) home life in harmony with work; (6) job satisfaction; (7) professional commitment; and (8) organizational commitment. Data for this study were obtained from the 2014 National Pharmacist Workforce Survey. For inclusion in analysis, respondents needed to report that they were practicing as a pharmacist. In addition, they needed to provide usable responses for both their percent time devoted to medication providing and to patient care services. This resulted in a total of 1191 responses for the analysis. Data were analyzed using cluster analysis, factor analysis, Cronbach coefficient alpha, chi-square analysis, ANOVA, and linear regression. The findings provide a description of pharmacists’ quality of work life in 2014 and show how type of work, variety of work, and work setting categories are associated with quality of work life for pharmacists.

## 1. Introduction

### 1.1. Quality of Work Life for Pharmacists

Pharmacists’ work environments and the amount and type of work performed can influence pharmacists’ quality of work life [1,2,3] and their level of work performance [4]. Pharmacist “quality of work life” research has investigated a variety of constructs such as job stress, level of control, job satisfaction, commitment to their profession and their employing organization, work motivation, and role overload [5,6,7,8,9,10,11,12,13,14,15,16,17,18,19,20,21,22,23,24,25,26,27,28,29,30,31,32,33,34,35,36,37,38,39]. These constructs have been associated with burnout, job turnover, and work performance and the findings have been used for improving pharmacist work systems, work processes, and patient safety [5,6,12,31,34]. As pharmacy work environments and the type of work performed by pharmacists change, it is useful to continually apprise pharmacists’ quality of work life. As these changes take place, an important question to consider is if pharmacists’ quality of work life is more closely associated with work setting category or with work activity type. That is, does quality of work life depend more on characteristics of the setting in which pharmacists work or on the types of activities in which they are engaged?

### 1.2. Study Objectives

The overall goal for this study was to use data from the most recently conducted National Pharmacist Workforce Survey (2014) to compare quality of work life dimensions for actively practicing pharmacists in the United States categorized by (1) work activity and (2) work setting. The eight components of quality of work life contained in the national survey were: (1) time stress; (2) responsibility stress; (3) level of control; (4) work in harmony with home life; (5) home life in harmony with work; (6) job satisfaction; (7) professional commitment; and (8) organizational commitment [5,6,7,8,9,10,11,12,13,14,15,16,17,18,19,20,21,22,23,24,25,26,27,28,29,30,31,32,33,34,35,36,37,38,39]. Pharmacists were placed into five segments based on proportion of time devoted to work activities [40,41] and were: (1) Medication Provider; (2) Medication Provider who also Provides Patient Care; (3) Patient Care Provider who also Provides Medication; (4) Patient Care Provider; and (5) Other Activity Pharmacist. The work setting segments of pharmacists were: (1) Community; (2) Hospital; (3) Other, Pharmacy and (4) Other, Non-Pharmacy [35]. Definitions and measures for each of these are described in the Methods section. 

## 2. Materials and Methods

### 2.1. Data Collection

Data for this study were obtained from the 2014 National Pharmacist Workforce Survey [35]. For that survey, a random sample of 5200 pharmacists was selected from a list that contained 250,652 unduplicated licensed individuals. A mailed questionnaire with multiple follow-ups was designed using principles from Dillman [42] in which a multiple-contact approach was utilized. Treatment of Human Subjects approval was reviewed and granted by the University of Minnesota Institutional Review Board.

### 2.2. Quality of Work Life Variables

Eight quality of work life variables were obtained from the 2014 National Pharmacist Workforce Survey [35] data file. The items were based on previous research [1,36,37,38,39]. Items used for measuring these constructs are presented in the Appendix A. For a complete summary of the development and composition of these variables, we refer interested readers to the Final Report of the 2014 National Sample Survey of the Pharmacist Workforce [35]. The constructs were:

Time Stress [36] defined as: harmful physical and emotional responses that occur when the time requirements of the job do not match the capabilities, resources, or needs of the worker. It was a seven-item measure with each item rated from 0 = not stressful at all to 3 = highly stressful.

Responsibility Stress [1] defined as: harmful physical and emotional responses that occur when the responsibilities of the job do not match the capabilities, resources, or needs of the worker. It was an eight-item measure with each item rated from 0 = not stressful at all to 3 = highly stressful.

Level of Control [1] defined as: ability of the worker to influence and make decisions about work place systems, processes, and policies. It was a six-item measure with each item rated from 0 = no control to 4 = total control.

Work in Harmony with Home Life [37] defined as: the extent to which the demands of work do not interfere with the worker’s home, family, or social life. It was a two-item measure with each item rated from 1 = strongly disagree to 7 = strongly agree.

Home Life in Harmony with Work [1] defined as: the extent to which the demands of home life do not interfere with the workers’ job- or career-related activities. It was a two-item measure with each item rated from 1 = strongly disagree to 7 = strongly agree.

Job Satisfaction [37] defined as: the level of contentment workers have with their job. It was a five-item measure with each item rated from 1 = very dissatisfied to 5 = very satisfied.

Professional Commitment [38] defined as: the individual’s psychological attachment to the pharmacy profession. It was a five-item measure with each item rated from 1 = strongly disagree to 5 = strongly agree.

Organizational Commitment [39] defined as: the individual’s psychological attachment to his or her work setting organization. It was a six-item measure with each item rated from 1 = strongly disagree to 7 = strongly agree.

### 2.3. Work Activity Segments

Schommer and colleagues [40,41], categorized pharmacists into five groups or segments via cluster analysis techniques based on the percent time spent in two categories of work activity: (1) medication providing and (2) patient care at their place of employment. These two work activities were defined in the survey as follows:

- (Medication Providing) Patient Care Services Associated with Medication Dispensing: preparing, distributing, and administering medication products, including associated consultation, interacting with patients about selection and use of over-the-counter products, and interactions with other professionals during the medication dispensing process.
- (Patient Care) Patient Care Services Not Associated with Medication Dispensing: assessing and evaluating patient medication-related needs, monitoring and adjusting patients’ treatments to attain desired outcomes, and other services designed for patient care management.

The five segments of pharmacists were: (1) Medication Provider; (2) Medication Provider who also Provides Patient Care; (3) Patient Care Provider who also Provides Medication; (4) Patient Care Provider; and (5) Other Activity Pharmacist. Medication Provider pharmacists reported spending a high proportion of their time in activities associated with dispensing, with relatively little time in patient care activities. Conversely, Patient Care pharmacists spent a high proportion of their time in those activities, with little time in medication dispensing activities. The Medication Provider/Patient Care and Patient Care/Medication Provider segments had more activity time diversity with proportional mixes of times in those activities consistent with the labels assigned to the segments. The Other Activity Pharmacist segment had relatively low percent times in both of the bifurcating activities. The five segments were used in this study so that it would build upon previous research [40,41].

### 2.4. Work Settings

Data collected in the 2014 National Pharmacist Workforce Survey [35] included work setting type. These were categorized into four groups for the purpose of this study using categorization schemes applied in prior research [1,13,22,27,29]. The four work setting types were:

- Community (independent, small chain, large chain, mass merchandiser, supermarket)
- Hospital (non-government hospital/health system, government hospital/health system)
- Other, Pharmacy (nursing home, long term care, health maintenance organization, nuclear, clinic-based, mail service, central fill, and home health/infusion pharmacies)
- Other, Non-Pharmacy (pharmacy benefit administration, academic, government administration, pharmaceutical industry, consulting companies, professional associations, and other organizations that were not licensed as a pharmacy)

### 2.5. Other Independent Variables Used for This Study

Other variables that were used for categorization and descriptive purposes included: (1) respondent age; (2) gender; (3) holding a PharmD degree; (4) having residency training; and (5) hours worked per week. These variables were operationalized as follows:

- Respondent age was reported in years and categorized by decades (30 or less, 31 to 40, 41 to 50, 51 to 60, 61 to 70, and over 70).
- Gender was reported as male or female.
- Holding a PharmD and having residency training were reported as yes or no.
- The variable “hours worked per week” was reported in hours per week and categorized by Bureau of Labor Statistics categories (less than 35, 35 to 39, 40, 41 to 48, 49 to 59, 60 and over).

### 2.6. Data Analysis

To assign respondents into the five “pharmacist work activity segments” their reported percent times spent in medication providing activities and patient care activities were utilized as variables for a two-step cluster analysis using IBM SPSS version 24 statistical software. The two-step cluster analysis uses a scalable cluster algorithm. The first step of the analysis is to ‘pre-cluster’ each case (record) into many small sub-clusters through a sequential clustering approach. The second step of the analysis is to ‘cluster the sub-clusters’ resulting from step one into the final cluster solution using an agglomerative hierarchical clustering method. The log-likelihood distance measure (a probability-based distance) is applied for each step of the analysis so that both continuous and categorical variables can be used if so desired.

In order to confirm discriminant validity for the eight “quality of work life” variables, factor analysis and Cronbach coefficient analyses were conducted. Exploratory factor analysis was used to investigate the underlying factor structure of the eight “quality of work life” constructs that were created using the 41 items in the survey (see Appendix A). Factor analysis helps one understand the structure of a correlation matrix. It helps categorize a relatively large number of variables into a few overall factors. In this study, varimax rotation was used for factor analysis to maintain orthogonality of factors and to minimize the number of variables that had high loadings on a factor. Only factors with eigenvalues greater than one were included in the factor solution. In addition, only items with factor loadings with absolute values greater than 0.40 on one, and only one, factor were included for identifying factors. After the factors were identified, each one was assessed for reliability using Cronbach coefficient alpha. 

Descriptions and associations among the study variables were made using Chi-Square Analysis, Analysis of Variance, and Linear Regression Analysis. For this study, significance was set at *p* < 0.01 to account for the multiple comparisons conducted and also the nature of the dependent variables of interest, which were constructs computed by summating survey items. Model fit statistics were not computed since the purpose of this study was not to build predictive models, but rather, to describe associations between certain variables. Model building would require inclusion of other factors. Standardized coefficients were used for making comparisons among variables and refer to how many standard deviations a dependent variable will change, per standard deviation increase in the predictor variable.

## 3. Results

In 2014, 5053 surveys were presumed to be delivered to a pharmacist. Of these, 2445 (48.4%) were returned. For inclusion in analysis, respondents needed to report that they were practicing as a pharmacist. In addition, they needed to provide usable responses for both their percent time devoted to medication providing and to patient care services. This resulted in a total of 1191 responses for the analysis. For a description of the characteristics for all pharmacists in the United States, we refer interested readers to the Final Report of the 2014 National Sample Survey of the Pharmacist Workforce [35].

### 3.1. Work Activity Segments

Cluster analysis resulted in five pharmacist work activity segments similar to those reported by Schommer and colleagues previously [40,41]. Table 1 shows that the distribution of U.S. practicing pharmacist survey respondents was Medication Provider (40%), Medication Provider who also Provides Patient Care (23%), Patient Care Provider who also Provides Medication (14%), Patient Care Provider (13%), and Other Activity Pharmacist (10%). A brief description of each segment is presented next with the findings also summarized in Table 1.

#### 3.1.1. Medication Providers

In 2014, 40% of practicing pharmacist respondents were in the Medication Providers segment. These pharmacists devoted an average of 84% of their time to medication providing and only 6% to patient care services. In 2014, 56% were female, 46% had a PharmD degree (with the others holding a BPharm degree), and 6% had residency training. This segment contributed the fewest hours worked per week of any segment and primarily worked in Community settings (65%).

#### 3.1.2. Medication Providers Who Also Provide Patient Care

In 2014, 23% of practicing pharmacist respondents were in the ‘Medication Providers who also Provide Patient Care’ segment. These pharmacists devoted an average of 60% of their time to medication providing and 19% to patient care services. In 2014, 60% were female, 49% had a PharmD degree, and 4% had residency training. In 2014, 62% worked in Community settings, 23% worked in Hospital settings, and 14% worked in ‘Other, Pharmacy’ settings.

#### 3.1.3. Patient Care Providers Who Also Provide Medication

This segment (14% of practicing pharmacist respondents) devoted an average of 37% of their time to medication providing and 39% to patient care services. In 2014, 65% were female, 55% held a PharmD degree, and 18% had residency training. In 2014, 62% worked in Hospital settings, 24% in Community settings, and 11% in ‘Other, Pharmacy’ settings.

#### 3.1.4. Patient Care Providers

In 2014, 13% of practicing pharmacist respondents were in the Patient Care Provider segment. These pharmacists devoted an average of 8% of their time to medication providing and 75% to patient care services. They were the youngest of the five segments, on average. In 2014, 70% were female, 67% had a PharmD degree, and 42% had residency training. In 2014, 57% worked in Hospital settings and 27% worked in ‘Other, Pharmacy’.

#### 3.1.5. Other Activity Pharmacists

In 2014, 10% of practicing pharmacist respondents were in the ‘Other Activity Pharmacists’ segment. These pharmacists devoted an average of 6% of their time to medication providing and 5% to patient care services as defined in this study. Most of their time was devoted to other activities such as business/organization management, research, education, and other health-system improvement activities. In 2014, 54% were female, 63% had a PharmD degree, and 32% had residency training. This segment contributed the most hours worked per week of any segment and 38% of this segment of pharmacists worked in ‘Other, Non- Pharmacy’ settings with another 32% working in a Hospital setting.

### 3.2. Work Settings

Table 2 shows that the distribution of U.S. practicing pharmacists by setting was Community Pharmacy (46%), Hospital Pharmacy (32%), Other, Pharmacy (15%), and Other, Non-Pharmacy (7%). A brief description of each category is presented next with the findings also summarized in Table 2.

#### 3.2.1. Community

In 2014, 46% of practicing pharmacist respondents worked in Community settings. These pharmacists devoted an average of 69% of their time to medication providing and 12% to patient care services. In 2014, 56% were female, 47% had a PharmD degree (with the others holding a BPharm degree), and 3% had residency training. Further categorizing by work activity segments, these community pharmacists were mostly comprised of Medication Providers (57%) and Medication Providers who also Provide Patient Care (31%).

#### 3.2.2. Hospital

In 2014, 32% of practicing pharmacist respondents worked in Hospital settings. These pharmacists devoted an average of 44% of their time to medication providing and 34% to patient care services. In 2014, 62% were female, 57% had a PharmD degree, and 29% had residency training. When grouped by work activity segments, these hospital pharmacists were mostly Patient Care Providers who also Provide Medication (27%), Medication Providers (24%) and Patient Care Providers (22%).

#### 3.2.3. Other, Pharmacy

In 2014, 15% of practicing pharmacists were in the ‘Other, Pharmacy’ segment. These pharmacists devoted an average of 49% of their time to medication providing and 28% to patient care services as defined in this study. In 2014, 64% were female, 55% had a PharmD degree, and 13% had residency training. With regard to work activity segments, these ‘Other, Pharmacy’ pharmacists were mostly Medication Providers (36%), Patient Care Providers (22%), and Medication Providers who also Provide Patient Care (21%).

#### 3.2.4. Other, Non-Pharmacy

This segment (7% of practicing pharmacists) devoted an average of 19% of their time to medication providing and 22% to patient care services. Most of their time was devoted to other activities such as business/organization management, research, education, and other health-system improvement activities. In 2014, 64% were female, 57% held a PharmD degree, and 30% had residency training. These pharmacists, working in ‘Other, Non-Pharmacy’ settings, were mostly in the Other Activity Pharmacists (51%) and Patient Care Providers (22%) work activity segments.

### 3.3. Quality of Work Life

Findings from factor analysis showed that each of the 41 items used to measure these eight constructs met our factor analysis criteria (loaded on a factor with an eigenvalue greater than one, exhibited a factor loading with an absolute value >0.40, and loaded on one and only one factor). Factor loadings were consistent with the measures as summarized in the Appendix A. Complete factor analysis results may be obtained from the corresponding author. 

Cronbach coefficient alpha (for measures of three or more items) and correlation coefficients (for two-item measures) supported measure reliability: Time Stress (7-items, alpha = 0.83), Responsibility Stress (8-items, alpha = 0.81), Level of Control (6-items, alpha = 0.84), Work in Harmony with Home Life (2-items, r = 0.55), Home Life in Harmony with Work (2-items, r = 0.58), Job Satisfaction (5-items, alpha = 0.93), Professional Commitment (5-items, alpha = 0.89), and Organizational Commitment (6-items, alpha = 0.88).

Table 3 and Table 4 summarize mean scores for each of the eight quality of life constructs for respondents categorized by work activity segments (Table 3) and by work setting (Table 4). Table 3 reveals that Responsibility Stress, Level of Control, Job Satisfaction, Professional Commitment and Organizational Commitment scores were significantly different among practicing pharmacist respondents categorized by work activity segments. Other Activity Pharmacists scored higher than other segments on Level of Control, Job Satisfaction, and Professional and Organizational Commitments. Patient Care Providers who did and did not provide medications scored higher than other segments on Responsibility Stress.

Table 4 shows that Time Stress, Level of Control, Work in Harmony with Home Life, Job Satisfaction, Professional Commitment, and Organizational Commitment were significantly different among practicing pharmacist respondents categorized by work setting. Community pharmacists scored higher than other work settings on Time Stress. ‘Other, Non-Pharmacy’ pharmacists scored higher on Level of Control, Job Satisfaction, and Professional and Organizational Commitments. Work was more in harmony with home life for ‘Other, Pharmacy’ and ‘Other, Non-Pharmacy’ work settings.

The results presented so far show that work activity segments and work setting categories are associated with varying components of pharmacists’ quality of work life. To help describe these effects further, linear regression analysis was conducted so that the associations between work activity segments and work settings with quality of work life could be described while controlling for pharmacist gender, age, and hours worked per week. Standardized coefficients were used for making comparisons among variables and refer to how many standard deviations a dependent variable will change, per standard deviation increase in the predictor variable. Findings from these analyses for each of the eight work life constructs are summarized next (see Table 5 and Table 6).

#### 3.3.1. Time Stress

Time Stress was associated with gender and work setting (Table 6) and not associated with work activity segment (Table 5). Females reported higher Time Stress than males when controlling for work setting. In addition, pharmacists working in Hospitals and ‘Other, Pharmacy’ work settings reported lower Time Stress scores in comparison with other settings. It is noteworthy that gender was not significant when controlling for work activity segment (Table 5).

#### 3.3.2. Responsibility Stress

Responsibility Stress was associated with gender and work activity segment (Table 5) and not associated with work setting (Table 6). Female respondents reported higher Responsibility Stress than males. In addition, pharmacists working in the ‘Patient Care Provider who also Provides Medications’ and ‘Patient Care Provider’ categories reported higher Responsibility Stress. It is noteworthy that gender still was significant when controlling for work setting (Table 6).

#### 3.3.3. Level of Control

Table 5 and Table 6 show that Level of Control was associated with gender, age, hours worked per week, work activity segment and work setting. Female respondents reported lower Level of Control than males in both analyses (Table 5 and Table 6). Also, pharmacists aged 51 to 70 reported lower Level of Control than other age groups. Pharmacists working 49 to 59 hours per week reported higher Levels of Control than other groups in both models (Table 5 and Table 6). The ‘Other Activity Pharmacist’ work activity segment reported higher Level of Control than other groups (Table 5). Pharmacists working in ‘Other, Non-Pharmacy’ settings reported higher Level of Control compared to other groups.

#### 3.3.4. Work in Harmony with Home Life

Work in Harmony with Home Life was associated with age, hours worked per week, and work setting (see Table 6), but not with work activity segment (see Table 5). Scores for this construct were lower for those aged 31 to 40 in both models (Table 5 and Table 6) and higher for those who worked less than 35 hours per week or exactly 40 h per week in both models (Table 5 and Table 6). Community work settings exhibited significantly lower scores compared with the other three work settings (Table 6).

#### 3.3.5. Home Life in Harmony with Work

Home Life in Harmony with Work was associated with age, hours worked per week, and work activity segment (Table 5), but not with work setting (Table 6). Those aged 31 to 40 reported lower scores than other age groups. Those who worked 49 to 59 h per week reported higher scores than other groups. Also, those in the ‘Patient Care Provider’ work activity segment reported lower scores than other groups (Table 5). It is noteworthy that age was significant in the model while controlling for work setting (Table 6).

#### 3.3.6. Job Satisfaction

Job Satisfaction was associated with age, work activity segment (Table 5), and work setting (Table 6). Responded pharmacists with ages over 70 reported higher Job Satisfaction compared with other groups. Two work activity segments reported higher Job Satisfaction (Other Activity Pharmacist and Patient Care Provider segments). Pharmacists working in Community settings reported lower Job Satisfaction than other groups (Table 6).

#### 3.3.7. Professional Commitment

Professional Commitment was associated with gender, age, work activity segment (Table 5) and work setting (Table 6). Female respondents reported higher Professional Commitment than males in both models (Table 5 and Table 6). When controlling for work activity segment (Table 5), those aged 41 to 50 reported lower Professional Commitment than other groups. Also, the ‘Other Activity Pharmacist’ work activity segment reported higher Professional Commitment than other groups (Table 5). For work settings, both Hospital and Other, Non-Pharmacy groups reported higher Professional Commitment than the other two groups (Community and Other, Pharmacy).

#### 3.3.8. Organizational Commitment

Organizational Commitment was associated with work activity segment (Table 5) and with work setting (Table 6). Controlling for gender, age, and hours worked per week, ‘Other Activity Pharmacist’ was the work activity segment with the highest Organizational Commitment compared with the other groups. Controlling for gender, age, and hours worked per week, the work setting with the highest Organizational Commitment was ‘Other, Non-Pharmacy’.

## 4. Discussion

The goal for this study was to use data from the most recent National Pharmacist Workforce Survey (2014) to compare eight components of quality of work life for actively practicing pharmacists in the United States categorized by (1) work activity segment and (2) work setting category. Comparisons among these groups could help address the question: “Is pharmacists’ quality of work life more closely associated with work setting category or with work activity segment?” That is, does quality of work life depend more on characteristics of the setting in which pharmacists work or on the types of activities in which they are engaged?

### 4.1. Differential Effects of Work Activity Segments and Work Setting Categories

The findings showed that work activity segments (and not work setting categories) were associated with responsibility stress and home life in harmony with work. For both of these quality of work life constructs, activities associated with patient care had unfavorable impacts. In addition, the findings showed that work setting categories (and not work activity segments) were associated with time stress and work in harmony with home life. For both of these quality of work life constructs, working in community pharmacy settings had unfavorable impacts. These findings are similar to an earlier study by Gaither [43] which indicated that work-home conflict and role overload (time stress) varied by practice setting. Finally, the findings showed that both work activity segments and work setting categories were associated with level of control, job satisfaction, professional commitment, and organizational commitment. 

Thus, we conclude that both the characteristics of the setting in which pharmacists work and the types of activities in which they are engaged are associated with various aspects of their quality of work life. The pattern of findings also showed that “Other Activity Pharmacists” and those working in “Other, Non-Pharmacy” settings may report more control, satisfaction and commitment to their profession and organization than other pharmacists, potentially because of the task variety they may experience compared to other types of pharmacists. This is conjecture on our part, but these findings may reveal the importance that task variety may have in quality of work life.

### 4.2. Implications for Pharmacy Practice

The findings showed that types of activities in which pharmacists are engaged (including task variety) and work setting categories can be useful for describing different components of quality of work life for practicing pharmacists. This has implications for pharmacy practice. For example, community pharmacy settings still are dominated by medication dispensing. However, as the work activities in community settings continue to evolve in the future [34], assessing quality of work life in community settings will benefit from segmenting the pharmacist workforce by their work activities. Furthermore, increasing variety in dispensing and patient care work activities for pharmacists may be a way to improve their quality of work life.

It is noteworthy that pharmacists in the Patient Care Provider segment reported higher levels of responsibility stress and lower levels of home life being in harmony with work. Nonetheless, they also reported higher job satisfaction than their medication provider counterparts. It is also noteworthy that pharmacists in the “other activity pharmacist” segment reported higher level of control, higher job satisfaction, higher professional commitment, and higher organizational commitment than their medication provider counterparts. These two groups of pharmacists still are relatively small segments of the overall practicing pharmacist population (13% and 10%, respectively), but appear to offer beneficial quality of work life characteristics. We believe that these are growing segments of the pharmacist workforce and are worthy of particular attention.

At the time this article is being written, health care organizations are engaged in mergers, acquisitions, partnerships, vertical integration and horizontal integration. We propose that this will create new work settings and new work activities (including task variety) for pharmacists; both of which will likely impact pharmacists’ quality of work life.

### 4.3. Recommendations for Future Research

Based on the findings, we recommend that future research would continue to describe pharmacists’ quality of work life using both work activity segments and work setting categories. Further, we propose that “task variety” will be an important variable to develop and study further. As health care organizations evolve and create new work settings and work activities for pharmacists, quality of work life research will continue to be relevant in these new systems of care. Within these new systems of care, we propose that application of the quality of work life constructs that were used in this study would not only apply to pharmacists, but also to other co-workers as well. The measurement of quality of work life for all workers within systems of care would provide new insights for improving work systems, work processes, and patient safety.

## 5. Limitations

The results and our interpretation of them should be tempered with the limitations of the study. The results are based on respondents’ self-reports, raising questions regarding the extent to which respondents gave socially desirable responses. Non-response bias is another limitation. It is possible that responders were more interested in the topic we studied or had stronger opinions about the questions we asked than those who chose not to respond. For our analysis, usable data from respondents working as pharmacists were used. While our findings are representative of actively practicing pharmacists, it should be noted that our analysis did not include licensed pharmacists who were outside of these domains (retired, unemployed, working in a pharmacy related field but not practicing as a pharmacist, or working outside of a pharmacy related field). For a complete summary for how the characteristics of pharmacists used in this study compare with characteristics of all pharmacists in the United States, we refer interested readers to the Final Report of the 2014 National Sample Survey of the Pharmacist Workforce [35]. Finally, patient care services may vary widely among responders in terms of specific activities and various roles served. This variable should be viewed as a broadly defined one when interpreting the findings.

## 6. Conclusions

Based on the times spent in medication dispensing and patient care activities, pharmacists were categorized into segments that reflected task and work effort variety. Segment membership, work setting, and other variables were examined for how they influence different dimensions of quality of work life. For some aspects of pharmacists’ quality or work life, variation in the time spent in different types of work activities and work setting influenced their levels of work life quality. Further monitoring of pharmacist quality of work life may yield additional insights to how both work activity and work setting are influential as contemporary practice changes and pharmacist roles evolve.

## Figures and Tables

**Table 1:** Description of U.S. Practicing Pharmacists by Work Activity Segments.

	Medication Provider	Medication Provider Who also Provides Patient Care	Patient Care Provider Who Also Provides Medication	Patient Care Provider	Other Activity Pharmacist	Overall
	N = 480	N = 275	N = 169	N = 150	N = 117	N = 1191
Proportion of Total Sample	40%	23%	14%	13%	10%	100%
Mean % Time Devoted to Medication Providing ANOVA *p* < 0.001	84%	60%	37%	8%	6%	54%
Mean % Time Devoted to Patient Care ANOVA *p* < 0.001	6%	19%	39%	75%	5%	22%
Mean Age (years) ANOVA *p* < 0.001	47.9	46.0	45.1	43.5	48.1	46.5
Female Gender (%) X^2^ *p* = 0.01	56%	60%	65%	70%	54%	60%
Hold PharmD (%) X^2^ *p* < 0.001	46%	49%	55%	67%	63%	52%
Residency Training (%) X^2^ *p* < 0.001	6%	4%	18%	42%	32%	15%
Mean Hrs Worked /Wk ANOVA *p* < 0.001	39.4	40.1	41.5	41.1	47.5	40.9
Current Work Setting X^2^ *p* < 0.001						
Community ^a^	65%	62%	24%	4%	13%	46%
Hospital	19%	23%	62%	57%	32%	32%
Other, Pharmacy ^b^	14%	14%	11%	27%	17%	15%
Other, Non-Pharmacy ^c^	3%	1%	4%	13%	38%	7%

^a^ “Community” included: independent, chain, mass merchandiser and supermarket pharmacies; ^b^ “Other, Pharmacy” included: nursing home, long term care, health maintenance organization, nuclear, clinic-based, mail service, central fill, and home health/infusion pharmacies; ^c^ “Other, Non-Pharmacy” included: pharmacy benefit administration, academic, government administration, pharmaceutical industry, consulting companies, professional associations, and other organizations that were not licensed as a pharmacy. Construct definitions and measurement items are presented in the Appendix A.

**Table 2:** Description of U.S. Practicing Pharmacists by Work Setting.

	Community ^a^	Hospital	Other, Pharmacy ^b^	Other, Non-Pharmacy ^c^	Overall
	N = 543	N = 380	N = 180	N = 88	N = 1191
Proportion of Total Sample	46%	32%	15%	7%	100%
Mean % Time Devoted to Medication Providing AVOVA *p* <0.001	69%	44%	49%	19%	54%
Mean % Time Devoted to Patient Care ANOVA *p* < 0.001	12%	34%	28%	22%	22%
Mean Age (years) ANOVA *p* = 0.60	46.2	46.3	47.2	47.6	46.5
Female Gender (%) X^2^ *p* = 0.13	56%	62%	64%	64%	60%
Hold PharmD (%) X^2^ *p* = 0.02	47%	57%	55%	57%	53%
Residency Training (%) X^2^ *p* < 0.001	3%	29%	13%	30%	15%
Mean Hrs Worked /Wk ANOVA *p* = 0.001	40.2	41.3	40.0	45.2	40.9
Work Activity Segment X^2^ *p* < 0.001					
Medication Provider	57%	24%	36%	15%	40%
Medication Provider who also Provides Patient Care	31%	17%	21%	5%	23%
Patient Care Provider who also Provides Medication	8%	27%	10%	7%	14%
Patient Care Provider	1%	22%	22%	22%	13%
Other Activity Pharmacist	3%	10%	11%	51%	10%

^a^ “Community” included: independent, chain, mass merchandiser and supermarket pharmacies; ^b^ “Other, Pharmacy” included: nursing home, long term care, health maintenance organization, nuclear, clinic-based, mail service, central fill, and home health/infusion pharmacies; ^c^ “Other, Non-Pharmacy” included: pharmacy benefit administration, academic, government administration, pharmaceutical industry, consulting companies, professional associations, and other organizations that were not licensed as a pharmacy. Construct definitions and measurement items are presented in the Appendix A.

**Table 3:** Quality of Work Life for U.S. Practicing Pharmacists by Work Activity Segments.

	Medication Provider	Medication Provider Who Also Provides Patient Care	Patient Care Provider Who Also Provides Medication	Patient Care Provider	Other Activity Pharmacist	Overall
	N = 480	N = 275	N = 169	N = 150	N = 117	N = 1191
Time Stress ANOVA *p* = 0.09	14.5 (4.5)	14.6 (4.6)	14.8 (3.9)	13.3 (4.6)	13.3 (4.8)	14.4 (4.5)
Responsibility Stress ANOVA *p* < 0.001	11.2 (4.7)	11.8 (4.5)	12.9 (4.3)	13.0 (5.1)	10.5 (4.7)	11.8 (4.7)
Level of Control ANOVA *p* < 0.001	9.7 (4.6)	10.3 (4.9)	10.3 (5.0)	9.4 (4.3)	15.0 (4.9)	10.3 (4.9)
Work In Harmony with Home Life ANOVA *p* = 0.30	7.6 (3.4)	7.3 (3.2)	7.2 (3.3)	7.8 (3.4)	7.7 (3.3)	7.5 (3.3)
Home Life in Harmony with Work ANOVA *p* = 0.02	11.7 (2.7)	11.5 (2.6)	11.3 (2.7)	11.3 (2.7)	11.0 (3.0)	11.4 (2.8)
Job Satisfaction ANOVA *p* < 0.001	16.2 (4.9)	16.5 (4.8)	17.0 (4.9)	17.7 (4.9)	19.2 (4.5)	16.9 (4.9)
Professional Commitment ANOVA *p* = 0.001	17.1 (5.3)	16.9 (5.3)	17.0 (5.1)	17.7 (5.3)	19.2 (4.7)	17.3 (5.2)
Organizational Commitment ANOVA *p* < 0.001	26.1 (9.0)	26.9 (8.7)	26.8 (8.7)	27.1 (8.7)	30.6 (7.8)	27.0 (8.8)

Construct definitions and measurement items are presented in the Appendix A. Table reports mean (s.d.).

**Table 4:** Quality of Work Life for U.S. Practicing Pharmacists by Work Setting.

	Community ^a^	Hospital	Other, Pharmacy ^b^	Other, Non-Pharmacy ^c^	Overall
	N = 543	N = 380	N = 180	N = 88	N = 1191
Time Stress ANOVA *p* < 0.001	15.1 (4.2)	13.2 (4.3)	13.3 (5.0)	13.2 (5.1)	14.4 (4.5)
Responsibility Stress ANOVA *p* = 0.34	11.7 (4.5)	12.1 (4.7)	11.6 (5.0)	10.8 (6.0)	11.8 (4.7)
Level of Control ANOVA *p* < 0.001	10.5 (4.7)	9.6 (4.6)	10.1 (5.7)	13.1 (5.0)	10.3 (4.9)
Work In Harmony with Home Life ANOVA *p* < 0.001	7.1 (3.2)	7.6 (3.3)	8.3 (3.4)	8.4 (3.4)	7.5 (3.3)
Home Life in Harmony with Work ANOVA *p* = 0.17	11.5 (2.8)	11.2 (2.8)	11.7 (2.6)	11.6 (2.8)	11.4 (2.8)
Job Satisfaction ANOVA *p* < 0.001	15.8 (4.9)	17.3 (4.8)	18.0 (4.5)	19.3 (3.9)	16.9 (4.9)
Professional Commitment ANOVA *p* < 0.001	16.8 (5.4)	17.8 (5.1)	16.8 (5.1)	19.7 (4.6)	17.3 (5.2)
Organizational Commitment ANOVA *p* = 0.002	26.4 (9.0)	27.0 (8.6)	27.2 (9.1)	30.4 (7.0)	27.0 (8.8)

^a^ “Community” included: independent, chain, mass merchandiser and supermarket pharmacies; ^b^ “Other, Pharmacy” included: nursing home, long term care, health maintenance organization, nuclear, clinic-based, mail service, central fill, and home health/infusion pharmacies; ^c^ “Other, Non-Pharmacy” included: pharmacy benefit administration, academic, government administration, pharmaceutical industry, consulting companies, professional associations, and other organizations that were not licensed as a pharmacy. Construct definitions and measurement items are presented in the Appendix A. Table reports mean (s.d.).

**Table 5:** Association of Gender, Age, Hours Worked per Week, and Work Activity Segments with Quality of Work Life for U.S. Practicing Pharmacists.

	Significant Variables	Significant Variable Categories	Standardized Coefficient	*p*-Value (if <0.01)
Time Stress	None			
Responsibility Stress	Gender	Male	(reference)	-
		Female	0.13	<0.001
	Work Activity Segment	Medication Provider	(reference)	
		Patient Care Provider + Medication	0.14	<0.001
		Patient Care Provider	0.12	0.002
Level of Control	Gender	Male	(reference)	-
		Female	−0.13	<0.001
	Age	30 or less	(reference)	-
		51 to 60	−0.20	<0.001
		61 to 70	−0.14	0.001
	Hours Worked Per Week	less than 35	(reference)	-
		49 to 59	0.13	<0.001
	Work Activity Segment	Medication Provider	(reference)	-
		Other Activity Pharmacist	0.25	<0.001
Work In Harmony with Home Life	Age	30 or less	(reference)	-
		31 to 40	−0.13	0.007
	Hours Worked Per Week	less than 35	(reference)	-
		35 to 39	−0.10	0.003
		41 to 48	−0.25	<0.001
		49 to 59	−0.23	<0.001
		60 and over	−0.23	<0.001
Home Life in Harmony with Work	Age	30 or less	(reference)	-
		31 to 40	−0.15	0.002
	Hours Worked Per Week	less than 35	(reference)	-
		49 to 59	0.10	0.006
	Work Activity Segment	Medication Provider	(reference)	-
		Patient Care Provider	−0.11	0.001
Job Satisfaction	Age	30 or less	(reference)	-
		over 70	0.09	0.004
	Work Activity Segment	Medication Provider	(reference)	-
		Patient Care Provider	0.11	0.001
		Other Activity Pharmacist	0.20	<0.001
Professional Commitment	Gender	Male	(reference)	-
		Female	0.13	<0.001
	Age	30 or less	(reference)	-
		41 to 50	−0.13	0.008
	Work Activity Segment	Medication Provider	(reference)	-
		Other Activity Pharmacist	0.12	<0.001
Organizational Commitment	Work Activity Segment	Medication Provider	(reference)	-
		Other Activity Pharmacist	0.16	<0.001

Quality of Work Life construct definitions and measurement items are presented in the Appendix A. Independent variables were operationalized for regression analysis as: Gender: Male (reference); Female. Age: 30 or less (reference); 31 to 40; 41 to 50; 51 to 60; 61 to 70; Over 70. Hours Worked Per Week: Less than 35 (reference); 35 to 39; 40; 41 to 48; 49 to 59; 60 and over. Work Activity Segment: Medication Provider (reference); Medication Provider who also Provides Patient Care; Patient Care Provider who also Provides Medication; Patient Care Provider; Other Activity Pharmacist. Standardized coefficients refer to how many standard deviations a dependent variable will change, per standard deviation increase in the predictor variable.

**Table 6:** Association of Gender, Age, Hours Worked per Week, and Work Setting with Quality of Work Life for U.S. Practicing Pharmacists.

	Significant Variables	Significant Variable Categories	Standardized Coefficient	*p*-Value (if <0.01)
Time Stress	Gender	Male	(reference)	-
		Female	0.11	0.009
	Work Setting	Community	(reference)	-
		Hospital	−0.19	<0.001
		Other, Pharmacy	−0.15	<0.001
Responsibility Stress	Gender	Male	(reference)	-
		Female	0.15	<0.001
Level of Control	Gender	Male	(reference)	-
		Female	−0.13	<0.001
	Age	30 or less	(reference)	-
		51 to 60	−0.17	0.001
		61 to 70	−0.12	0.004
	Hours Worked Per Week	less than 35	(reference)	-
		49 to 59	0.17	<0.001
	Work Setting	Community	(reference)	-
		Other, Non-Pharmacy	0.12	<0.001
Work In Harmony with Home Life	Age	30 or less	(reference)	-
		31 to 40	−0.13	0.006
	Hours Worked Per Week	less than 35	(reference)	-
		35 to 39	−0.10	0.002
		41 to 48	−0.25	<0.001
		49 to 59	−0.24	<0.001
		60 and over	−0.23	<0.001
	Work Setting	Community	(reference)	-
		Hospital	0.08	0.009
		Other, Pharmacy	0.13	<0.001
		Other, Non-Pharmacy	0.13	<0.001
Home Life in Harmony with Work	Age	30 or less	(reference)	-
		31 to 40	−0.16	0.001
Job Satisfaction	Age	30 or less	(reference)	-
		over 70	0.09	0.002
	Work Setting	Community	(reference)	-
		Hospital	0.16	<0.001
		Other, Pharmacy	0.16	<0.001
		Other, Non-Pharmacy	0.20	<0.001
Professional Commitment	Gender	Male	(reference)	-
		Female	0.12	<0.001
	Work Setting	Community	(reference)	-
		Hospital	0.10	0.002
		Other, Non-Pharmacy	0.13	<0.001
Organizational Commitment	Work Setting	Community	(reference)	-
		Other, Non-Pharmacy	0.12	<0.001

Quality of Work Life construct definitions and measurement items are presented in the Appendix A. Independent variables were operationalized for regression analysis as: Gender: Male (reference); Female. Age: 30 or less (reference); 31 to 40; 41 to 50; 51 to 60; 61 to 70; Over 70. Hours Worked Per Week: Less than 35 (reference); 35 to 39; 40; 41 to 48; 49 to 59; 60 and over. Work Setting: Community (reference); Hospital; Other, Pharmacy Setting; Other, Setting Non-Pharmacy. “Community” included: independent, chain, mass merchandiser and supermarket pharmacies. “Other, Pharmacy” included: nursing home, long term care, health maintenance organization, nuclear, clinic-based, mail service, central fill, and home health/infusion pharmacies. “Other, Non-Pharmacy” included: pharmacy benefit administration, academic, government administration, pharmaceutical industry, consulting companies, professional associations, and other organizations that were not licensed as a pharmacy. Standardized coefficients refer to how many standard deviations a dependent variable will change, per standard deviation increase in the predictor variable.

## Appendix A. Quality of Work Life Variables

### Definitions and Measurement Items

**Time Stress**—harmful physical and emotional responses that occur when the time requirements of the job do not match the capabilities, resources, or needs of the worker. Seven-item measure with each item rated from 0 = not stressful at all to 3 = highly stressful. Possible range of scores: 0 to 21; scale midpoint = 10.5. Cronbach coefficient alpha = 0.83. In general, how stressful is:

- Being interrupted by phone calls for people while performing job duties?
- Not being staffed with an adequate number of pharmacists?
- Not being staffed with an adequate number of technicians?
- Doing excessive paper work or documentation (i.e., third party work, patient records)?
- Having to meet quotas set by management?
- Having so much work to do that everything cannot be done well?
- Dealing with difficult patients?

**Responsibility Stress**—harmful physical and emotional responses that occur when the responsibilities of the job do not match the capabilities, resources, or needs of the worker. Eight-item measure with each item rated from 0 = not stressful at all to 3 = highly stressful. Possible range of scores: 0 to 24; scale midpoint = 12. Cronbach coefficient alpha = 0.81. In general, how stressful is:

- Learning to use new technology or automation?
- Dealing with difficult coworkers?
- Disagreeing with other health care professionals concerning the treatment of patients?
- Keeping up with new developments in order to maintain professional competency?
- Possessing inadequate information regarding a patient’s medical condition?
- Feeling ultimately responsible for patient outcomes from drug therapy?
- Fearing that I will make a mistake in treating a patient?
- Delegating previous or new tasks to pharmacy technicians?

**Level of Control**—ability of the worker to influence and make decisions about work place systems, processes, and policies. Six-item measure with each item rated from 0 = no control to 4 = total control. Possible range of scores: 0 to 24; scale midpoint = 12. Cronbach coefficient alpha = 0.84. At your practice site, how much control do you have over:

- Your ability to take time from work for non-work activities?
- The development of workplace policies?
- The responsibilities delegated to support staff?
- How workplace problems are solved?
- The time spent in various work activities?
- The quality of care provided to patients?

**Work in Harmony with Home Life**—the extent to which the demands of work do not interfere with the worker’s home, family, or social life. Two-item measure with each item rated from 1 = strongly disagree to 7 = strongly agree. Possible range of scores: 2 to 14; scale midpoint = 8. Pearson correlation coefficient = 0.55.

- In general, the demands of work do not interfere with my home, family or social life.
- In general, my work has disadvantages for my home, family, or social life (reverse coded).

**Home Life in Harmony with Work**—the extent to which the demands of home life do not interfere with the workers’ job or career related activities. Two-item measure with each item rated from 1 = strongly disagree to 7 = strongly agree. Possible range of scores: 2 to 14; scale midpoint = 8. Pearson correlation coefficient = 0.58.

- Often, my home, family or social life interferes with my responsibilities at work, such as getting to work on time, accomplishing daily work tasks, or working overtime (reverse coded).
- Often, my home, family or social life keeps me from spending the amount of time I would like to spend on job or career-related activities (reverse coded).

**Job Satisfaction**—the level of contentment workers have with their job. Five-item measure with each item rated from 1 = very dissatisfied to 5 = very satisfied. Possible range of scores: 5 to 25; scale midpoint = 15. Cronbach coefficient alpha = 0.93. In general, how satisfied are you with:

- Your present job when compared to jobs in other organizations?
- The progress you are making toward the goals you set?
- The chance your job gives you to do what you are best at doing?
- Your present job in light of your career expectations?
- Your present job when you consider the expectations you had when you took the job?

**Professional Commitment**—the individual’s psychological attachment to the pharmacy profession. Five-item measure with each item rated from 1 = strongly disagree to 5 = strongly agree. Possible range of scores: 5 to 25; scale midpoint = 15. Cronbach coefficient alpha = 0.89.

- If I could do it all over again, I would not choose to work in the pharmacy profession (reverse coded).
- For me, this is the ideal profession for a life’s work.
- I am disappointed that I ever entered the pharmacy profession (reverse coded).
- I like this profession too well to give it up.
- If I could go into a different profession other than pharmacy which paid the same, I would probably do so (reverse coded).

**Organizational Commitment**—the individual’s psychological attachment to his or her work setting organization. Six-item measure with each item rated from 1 = strongly disagree to 7 = strongly agree. Possible range of scores: 6 to 42; scale midpoint = 24. Cronbach coefficient alpha = 0.88.

- I do not feel like “part of the family” at my organization (reverse coded).
- I do not feel “emotionally attached” to this organization (reverse coded).
- This organization has a great deal of personal meaning for me.
- I do not feel a strong sense of belonging to my organization (reverse coded).
- There is a high level of trust between top management and staff.
- There is a high level of trust between pharmacists/pharmacy staff and other health care providers.
